# Association Between Myocardium Hypertrophy And Dementia: A Population Based Autopsy Study

**DOI:** 10.1002/alz.089688

**Published:** 2025-01-09

**Authors:** Heberti Eliakim Silva, Regina Silva Paradela, Renata Elaine Paraizo Leite, Carlos Augusto Pasquallucci, Lea T. Grinberg, Ricardo Nitrini, Wilson Jacob‐Filho, Claudia Kimie Suemoto

**Affiliations:** ^1^ University of São Paulo Medical School, São Paulo, São Paulo Brazil; ^2^ Biobank for Aging Studies of the University of São Paulo Medical School, São Paulo Brazil; ^3^ Physiopathology in Aging Laboratory (LIM‐22), Department of Internal Medicine, University of Sao Paulo Medical School, São Paulo, São Paulo Brazil; ^4^ Biobank for aging studies of the University of São Paulo, São Paulo Brazil; ^5^ UCSF Alzheimer’s Disease Research Center, San Francisco, CA USA; ^6^ Medical School of University of São Paulo, São Paulo Brazil; ^7^ Division of Geriatrics, Department of Internal Medicine, University of Sao Paulo Medical School, São Paulo, São Paulo Brazil

## Abstract

**Background:**

Hypertension is a midlife modifiable risk factor for dementia. Uncontrolled hypertension is related to brain damage that could result in cognitive impairment, and one way of evaluating uncontrolled hypertension through life is assessing left ventricular hypertrophy (LVH). Previous imaging studies have shown a relationship between LVH and cognitive impairment. However, no previous study has evaluated this association using direct measurements obtained through autopsy. Furthermore, little is known about this relationship in diverse populations in low and middle‐income countries like Brazil, where the prevalence of dementia is expected to be higher than in high‐income countries. This study aimed to evaluate the association between LVH and dementia using data from an autopsy study in an admixed sample.

**Method:**

We used data from the Biobank for Aging Studies of the University of São Paulo Medical School. The diagnosis of hypertension was obtained through a structured interview with an informant close to the deceased. The LVH was defined based on anatomical analysis and the left ventricular free wall measurement. Cognitive abilities were evaluated by the Clinical Dementia Rating Scale sum of boxes. The analyses were performed using regression models adjusted for sociodemographic and clinical confounders.

**Result:**

In 1067 participants (mean age 70.6±11.9 years old, mean education 4.6±3.8 years, 46.2% women, 66.4% White), LVH was not associated with cognitive abilities in multivariate analyses adjusted for aged, education, sex, race, diabetes, heart disease, body mass index, smoking, alcohol use, and physical inactivity. Age, sex, education, and race did not modify this relationship.

**Conclusion:**

We did not find an association between LVH and cognitive impairment in our sample. Further analyses will be important to investigate the possibility of attrition bias.

